# High‐intensity training in normobaric hypoxia enhances exercise performance and aerobic capacity in Thoroughbred horses: A randomized crossover study

**DOI:** 10.14814/phy2.14442

**Published:** 2020-05-22

**Authors:** Kazutaka Mukai, Hajime Ohmura, Akira Matsui, Hiroko Aida, Toshiyuki Takahashi, James H. Jones

**Affiliations:** ^1^ Sports Science Division Equine Research Institute Japan Racing Association Utsunomiya Tochigi Japan; ^2^ Department of Surgical and Radiological Sciences School of Veterinary Medicine University of California Davis CA USA; ^3^ Present address: Sports Science Division Equine Research Institute Japan Racing Association Shimotsuke Tochigi Japan; ^4^ Present address: Equine Science Division Hidaka Training and Research Center Japan Racing Association Urakawa Hokkaido Japan; ^5^ Present address: Equestrian Affairs Japan Racing Association Tokyo Japan

**Keywords:** aerobic capacity, horse, hypoxic training, performance

## Abstract

We examined the effects of high‐intensity training in normobaric hypoxia on aerobic capacity and exercise performance in horses and the individual response to normoxic and hypoxic training. Eight untrained horses were studied in a randomized, crossover design after training in hypoxia (HYP; 15.0% inspired O_2_) or normoxia (NOR; 20.9% inspired O_2_) 3 days/week for 4 weeks separated by a 4‐month washout period. Before and after each training period, incremental treadmill exercise tests were performed in normoxia. Each training session consisted of 1 min cantering at 7 m/s and 2 min galloping at the speed determined to elicit maximal oxygen consumption ( $\dot{V}$O_2_max) in normoxia. Hypoxia increased significantly more than NOR in run time to exhaustion (HYP, +28.4%; NOR, +10.4%, *p* = .001), $\dot{V}$O_2_max (HYP, +12.1%; NOR, +2.6%, *p* = .042), cardiac output ( $\dot{Q}$; HYP, +11.3%; NOR, −1.7%, *p* = .019), and stroke volume (*SV*) at exhaustion (HYP, +5.4%; NOR, −5.5%, *p* = .035) after training. No significant correlations were observed between NOR and HYP for individual changes after training in run time (*p* = .21), $\dot{V}$O_2_max (*p* = .99), $\dot{Q}$ (*p* = .19), and *SV* (*p* = .46) at exhaustion. Arterial O_2_ saturation during exercise in HYP was positively correlated with the changes in run time (*r* = .85, *p* = .0073), $\dot{Q}$ (*r* = .72, *p* = .043) and *SV* (*r* = .77, *p* = .026) of HYP after training, whereas there were no correlations between these parameters in NOR. These results suggest that high‐intensity training in normobaric hypoxia improved exercise performance and aerobic capacity of horses to a greater extent than the same training protocol in normoxia, and the severity of hypoxemia during hypoxic exercise might be too stressful for poor responders to hypoxic training.

## Graphical abstract

The present study demonstrates that high‐intensity training in hypoxia improves exercise performance and aerobic capacity of horses to a greater extent than the same training protocol in normoxia. However, the severity of hypoxemia during hypoxic exercise might be too stressful for poor responders to hypoxic training.

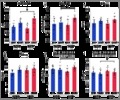

## 1 INTRODUCTION

Altitude training has been commonly used for elite athletes to improve their endurance performance since the Mexico City Olympic Games in 1968, and many altitude training protocols have been proposed (Millet, Roels, Schmitt, Woorons, & Richalet, 2010; Wilber, 2007). Live high‐train high (LHTH), live high‐train low (LHTL), and live low‐train high (LLTH) altitude trainings are three major training regimens that are commonly used and many researchers have demonstrated the effects of these altitude training protocols (Millet et al., 2010; Wilber, 2007; Wilber, Stray‐Gundersen, & Levine, 2007). In LLTH training, athletes live in normoxic conditions and train in a natural or simulated hypoxic environment. In theory, the stimulus of hypoxic exposure, in addition to training, will enhance the training adaptations experienced with normoxic training and will lead to greater improvements in performance (Dufour et al., 2006). The systematic reductions in O_2_ saturation (*S*O_2_) and/or O_2_ partial pressure (*P*O_2_) during the training may trigger various biochemical and structural changes in the skeletal muscle and metabolism that support oxidative processes (Melissa, MacDougall, Tarnopolsky, Cipriano, & Green, 1997; Terrados, Jansson, Sylven, & Kaijser, 1990; Zoll et al., 2006). Some authors suggest that LLTH may also improve anaerobic exercise performance (Hendriksen & Meeuwsen, 2003), possibly via increases in muscle buffering capacity (Gore et al., 2001) and increased glycolytic enzyme activity (Puype, Van Proeyen, Raymackers, Deldicque, & Hespel, 2013). However, a number of researchers have failed to demonstrate improvements in sea‐level performance after LLTH (Morton & Cable, 2005; Roels, Bentley, Coste, Mercier, & Millet, 2007; Truijens, Toussaint, Dow, & Levine, 2003). These conflicting reports on the effects of LLTH in the literature may be due to methodological differences, including the intensity of the hypoxic stimulus and the intensity, volume and duration of training in the hypoxic environment, as well as the individual's variability in adaptive response to hypoxia (Chapman, 2013).

Thoroughbred horses have high maximal oxygen consumption, and aerobic contribution to total energy expenditure in Thoroughbred horses is estimated to reach >70% for a 120‐s sprint (Eaton, Evans, Hodgson, & Rose, 1995; Ohmura et al., 2010), so that the improvement in aerobic capacity is inevitable for enhancing racing performance. The spleen releases stored erythrocytes at the onset of exercise in horses (Wagner et al., 1995) to increase the number of erythrocytes in the blood during exercise; the hematocrit of horses increases to nearly 60% during exercise (Mukai et al., 2008; Mukai, Hiraga, Takahashi, Ohmura, & Jones, 2010). Therefore, there are arguments that an increase in the number of circulating erythrocytes and hemoglobin caused by altitude training may not result in an additional benefit in aerobic capacity and exercise performance because maximal cardiac output may decline due to the increased blood viscosity above the optimal hematocrit (Boning, Maassen, & Pries, 2011). However, very few reports exist on the effects of altitude/hypoxic training in horses (Davie et al., 2017; Ohmura, Mukai, Takahashi, Takahashi, & Jones, 2017).

The hypothesis of this study was to determine if high‐intensity training in normobaric hypoxia would enhance aerobic capacity and exercise performance than normoxic training. A secondary hypothesis was to determine how much variability exists between individual horses to normoxic and hypoxic training in Thoroughbred horses.

## 2 MATERIALS AND METHODS

Protocols for the study were reviewed and approved by the Animal Welfare and Ethics Committee of the Japan Racing Association (JRA) Equine Research Institute (Permit number: 2013‐1, 2014‐1). All surgery was performed under sevoflurane anesthesia and all incisions for catheter placements were performed under local anesthesia using lidocaine. All efforts were made to minimize animal suffering.

### 2.1 Horses

Eight Thoroughbreds (five geldings, three females; mean ± *SD* age, 6.5 ± 1.7 years; body weight, 502 ± 14 kg at the onset of the study) were used in this study. The horses had a carotid artery surgically moved from the carotid sheath to a subcutaneous location under sevoflurane anesthesia to facilitate arterial catheterization. After recovery from surgery, the horses were trained to run on a treadmill (Sato I, Sato AB, Uppsala, Sweden) while wearing an open‐flow mask (Pascoe et al., 1999). After the surgery, horses were kept in 2‐ha pastures for approximately 6 hr/day every day for at least 4 months before treadmill experiments began.

### 2.2 Experimental design

**FIGURE 1:**
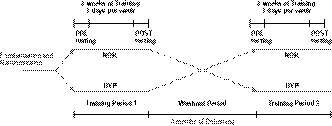
Experimental study design. In a randomized, crossover design, horses were trained in hypoxia (HYP; 15.0% inspired O_2_) or normoxia (NOR; 20.9% inspired O_2_) for 3 days/week for 4 weeks. Each training period was separated by 4 months to ensure a sufficient detraining interval

In a randomized crossover design, horses were trained in hypoxia (HYP; 15.0% inspired O_2_) or normoxia (NOR; 20.9% inspired O_2_) for 3 days/week on a treadmill inclined at a 6% grade and were pastured in 2‐ha pastures for approximately 6 hr/day on the other 4 days for 4 weeks. Each training period was separated by 4 months to ensure a sufficient detraining interval (Figure 1). The training session consisted of a warm‐up (walking at 1.7 m/s for 1 min and trotting at 4 m/s for 2 min), cantering at 7 m/s for 1 min, and at the speed previously determined to elicit 100% $\dot{V}$O_2_max measured in normoxia for 2 min, followed by a cool‐down (1.7 m/s for 3 min) in both groups.

### 2.3 Incremental exercise tests

Incremental exercise tests in normoxia were conducted before (PRE) and after (POST) the training period. The procedure for the incremental exercise test, including oxygen consumption measurements and blood sampling, has been described previously (Mukai et al., 2017). Briefly, after catheters and transducers were connected and tested, the horse began its exercise. The horse warmed up by trotting at 4 m/s for 3 min, then cantering or galloping up a 6% incline for 2 min each at 1.7, 4, 6, 8, 10, 12, 13, and 14 m/s until the horse could not maintain its position at the front of the treadmill with humane encouragement. This was defined as exhaustion. Run time to exhaustion was measured with a stopwatch. For each speed, the horse ran on the treadmill for 90 s to allow the O_2_ transport system to come to steady‐state (equine $\dot{V}$O_2_ comes to steady‐state faster than human does human $\dot{V}$O_2_), then $\dot{V}$O_2_ was calculated for the final 30 s of each step. Heart rate was recorded using a commercial heart rate monitor (S810, Polar, Kempele, Finland) and mean heart rate was calculated for the final 30 s of each step.

### 2.4 Oxygen consumption

Horses wore an open‐flow mask on the treadmill through which a rheostat‐controlled blower drew air. Air flowed through 25‐cm diameter tubing and across a pneumotachograph (LF‐150B, Vise Medical, Chiba, Japan) connected to a differential pressure transducer (TF‐5, Vise Medical, Chiba, Japan); this was done to ensure that bias flows during measurements were identical to those used during calibrations. Bias flow was set to keep changes in O_2_ concentration and CO_2_ concentrations <1.5% to avoid having the horses rebreathe CO_2_. Oxygen and CO_2_ concentrations were measured with an O_2_ and CO_2_ analyzer (MG‐360, Vise Medical, Chiba, Japan), and calibrations were used to calculate rates of O_2_ consumption and CO_2_ production with mass flow meters (CR‐300, Kofloc, Kyoto, Japan) using the N_2_‐dilution/CO_2_‐addition mass‐balance technique (Fedak, Rome, & Seeherman, 1981). Gas analyzer, thermohygrometer, and mass flowmeter outputs were also recorded on personal computers using commercial hardware and software (DI‐720 and Windaq Pro+, DATAQ, Akron, OH) sampling at 200 Hz.

### 2.5 Blood sampling

Before leading a horse onto the treadmill, an 18‐gauge catheter (Surflow, Terumo, Tokyo, Japan) was placed in the horse's carotid artery, and an 8‐F introducer (MO95H‐8, Baxter International, Deerfield, IL) in the jugular vein. A Swan‐Ganz catheter (SP5107U, Becton, Dickinson and Company, Franklin Lakes, NJ) was passed via the jugular vein so that its tip was positioned in the pulmonary artery, confirmed by measuring pressure at its tip with a pressure transducer (P23XL, Becton, Dickinson and Company, Franklin Lakes, NJ). Mixed‐venous blood samples were drawn from the tip of the Swan‐Ganz catheter and arterial samples from the 18‐gauge carotid catheter at timed intervals into heparinized syringes for the final 30 s of each step and 1, 3, 5, and 10 min after exhaustion, and stored on ice until measured immediately following the experiment. Blood samples were analyzed with a blood gas analyser (ABL800 FLEX, Radiometer, Copenhagen, Denmark) and for *S*O_2_ and O_2_ concentration (*C*O_2_) with a hemoximeter (ABL80 FLEX‐CO‐OX, Radiometer, Copenhagen, Denmark). Following measurement of blood gases and oximetry, the blood was sampled for plasma lactate concentration with a lactate analyzer (Biosen S‐Line, EKF‐diagnostic GmbH, Barleben, Germany) after being centrifuged at 1,870*g* for 10 min. The Swan‐Ganz catheter in the pulmonary artery was connected to a cardiac output computer (COM‐2, Baxter International, Deerfield, IL) so that its thermistor registered pulmonary arterial temperature, which was recorded at each blood sampling and used to correct the blood gas measurements.

### 2.6 Hypoxic training protocol and measurements during exercise in the first week of each training period

The procedure for producing the hypoxic condition was slightly modified from the method previously described (Ohmura et al., 2010). Briefly, a mixing chamber was connected to the upstream flexible tube on a 25‐cm diameter open‐flow mask through which a flow of N_2_ was blown into the upstream end of the flow system and mixed with a bias‐flow of air of 80–120 L/s to create the desired inspired O_2_ concentration. Nitrogen gas flow was controlled with a mass flow meter (Model DPM3, Kofloc, Kyoto, Japan) connected to compressed gas cylinders through a gas manifold. Nitrogen gas flow was adjusted to maintain 15% O_2_ by monitoring the O_2_ concentration in the downstream arm of the mass flow meter with an O_2_ analyzer (LC‐240UW, Vise Medical, Chiba, Japan) when horses ran in hypoxia.

In the first week of training for both groups, we collected arterial and mixed‐venous blood samples in the final 30 s of galloping at 100% $\dot{V}$O_2_max to measure blood gas variables and plasma lactate concentration, and we also recorded heart rate during galloping.

### 2.7 Statistical analysis

Data are presented as mean ± *SD*. The differences of within‐subject sums of the results from both periods were analyzed by an unpaired *t* test as a pretest to check carryover effects as described previously (Wellek & Blettner, 2012). After the pretest, the within‐subject differences of the results from period 1 and period 2 were analyzed by an unpaired *t* test to test for differences between training (Wellek & Blettner, 2012). Pearson correlation and a Benjamini–Hochberg procedure was used to determine the relationship between the changes in the variables after NOR and HYP and between the changes in the variable after training and *S*_a_O_2_ and plasma lactate concentration during training sessions. Differences in the variables during training sessions between NOR and HYP were assessed using a paired *t* test. Statistical analyses were performed with commercial software (JMP 13.1.0, SAS Institute Inc., Cary, NC) with significance defined as *p* < .05.

## 3 RESULTS

### 3.1 Heart rate, plasma lactate concentration, and blood gas variables during training sessions

**FIGURE 2:**
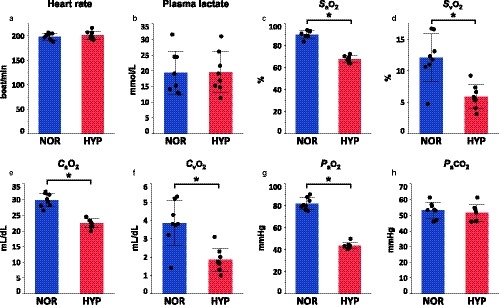
Heart rate, plasma lactate concentration, and blood gas variables during training sessions. Heart rate (a), plasma lactate concentration (b), arterial O_2_ saturation (*S*_a_O_2_, c), mixed‐venous O_2_ saturation (*S*_v_O_2_, d), arterial O_2_ concentration (*C*_a_O_2_, e), mixed‐venous O_2_ concentration (*C*_v_O_2_, f), arterial O_2_ partial pressure (*P*_a_O_2_, g), and arterial carbon dioxide partial pressure (*P*_a_CO_2_, h) in the final 30‐s of a 2‐min gallop at the speed eliciting 100% *V*O_2_max during training sessions either in normoxia (NOR; blue) or hypoxia (HYP; red). Values are means ± *SD* for eight horses. *Significantly different from NOR (*p* < .05)

Arterial O_2_ saturation (*S*_a_O_2_, *p* < .001), mixed‐venous O_2_ saturation (*S*_v_O_2_,* p* < .001), arterial O_2_ concentration (*C*_a_O_2_, *p* < .001), mixed‐venous O_2_ concentration (*C*_v_O_2_, *p* = .0011), and arterial O_2_ partial pressure (*P*_a_O_2_, *p* < .001) of HYP in the last 15 s of a 2‐min gallop were lower than those of NOR (Figure 2). There were no differences in heart rate, plasma lactate concentration nor arterial carbon dioxide partial pressure (*P*_a_CO_2_) in the last 15 s of a 2‐min gallop between the two groups (heart rate, *p* = .50; plasma lactate concentration, *p* = .96; *P*_a_CO_2_, *p = *.49, Figure 2).

### 3.2 Effects of normoxic and hypoxic training on performance and aerobic capacity

**FIGURE 3:**
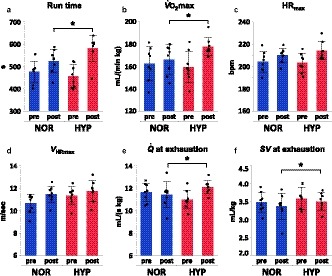
Effects of normoxic and hypoxic training on exercise performance and aerobic capacity. Run time (a), maximal oxygen consumption ( $\dot{V}$O_2_max, b), maximal heart rate (HR_max_, c), speed eliciting maximal heart rate (*V*_HRmax_, d), cardiac output ( $\dot{Q}$, e), and cardiac stroke volume (*SV*, F) at exhaustion before (PRE) and after (POST) training either in normoxia (NOR; blue) or hypoxia (HYP; red). Values are means ± *SD* for eight horses. *Significantly different from NOR (*p* < .05)

**FIGURE 4:**
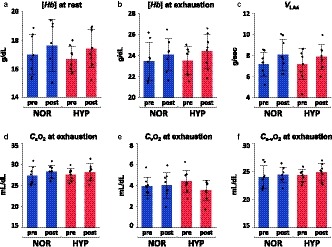
Effects of normoxic and hypoxic training on blood gas parameters and lactate threshold. Hemoglobin concentration ([*Hb*]) at rest (a) and at exhaustion (b), speed at which plasma lactate concentration reached 4‐mmol/l (*V*_LA4_, c), arterial O_2_ concentration (*C*_a_O_2_, d), mixed‐venous O_2_ concentration (*C*_v_O_2_, e), and arterial‐(mixed‐venous) O_2_ concentration difference (*C*_a‐v_O_2_, f) at exhaustion before (PRE) and after (POST) training either in normoxia (NOR; blue) or hypoxia (HYP; red). Values are means ± *SD* for eight horses. *Significantly different from NOR (*p* < .05)

There were no statistical differences in the PRE values between NOR and HYP for any parameters (Table 1), suggesting that the horses were successfully detrained during the washout period between the training periods. Hypoxia increased to a greater extent than did NOR in run time to exhaustion (HYP, +28.4%; NOR, +10.4%,* p* = .001), $\dot{V}$O_2_max (HYP, +12.1%; NOR, +2.6%, *p* = .042), cardiac output ( $\dot{Q}$) (HYP, +11.3%; NOR, −1.7%,* p* = .019), cardiac stroke volume (*SV*) at exhaustion (HYP, +5.4%; NOR, −5.5%; *p* = .035), and *LA*_peak_ (HYP, +41.4%; NOR, +1.3%, *p* = .025) (Figure 3, Table 1). There were no differences between the two groups after training in hemoglobin concentration ([*Hb*]) at rest (*p* = .87) nor at exhaustion (*p* = .55), maximal heart rate (HYP, +5.3%; NOR, +3.1%; *p* = .32), *C*_a_O_2_ (*p* = .68), *C*_v_O_2_ (*p* = .20) or arterial‐(mixed‐venous) O_2_ concentration difference (*C*_a‐v_O_2_, *p* = .69) at exhaustion, nor the speed at which plasma lactate concentration reached 4 mmol/l (*V*_LA4_, *p* = .71), nor the speed eliciting maximal heart rate (*V*_HRmax_, *p* = .24) (Figure 4, Table 1).

**FIGURE 5:**
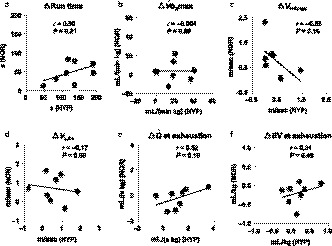
Correlations of individual responses after 4 weeks of normoxic and hypoxic training. Relationships between individual responses in run time (a), maximal oxygen consumption ( $\dot{V}$O_2_max, b), speed eliciting maximal heart rate (*V*_HRmax_, c), speed at which plasma lactate concentration reached 4‐mmol/l (*V*_LA4_, d), cardiac output ( $\dot{Q}$, e), and cardiac stroke volume (*SV*, f) at exhaustion

**FIGURE 6:**
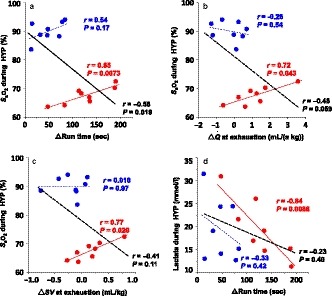
Correlations between the changes in run time, cardiac output ( $\dot{Q}$), and cardiac stroke volume (*SV*) after 4 weeks of hypoxic training and *S*_a_O_2_ (run time, a; $\dot{Q}$, b; *SV*, c) and plasma lactate concentration (d) during exercise in NOR (blue) and HYP (red). Solid regression lines for HYP (red) and total (black) are significant (*p* < .05) and dot regression lines for NOR (blue) and total (black) are not significant (*p* > .05)

No significant relationships were observed between NOR and HYP for individual changes after training in run time (*r* = .50, *p* = .21), $\dot{V}$O_2_max (*r* = −.004, *p* = .99), *V*_HRmax_ (*r* = −.55, *p* = .16), *V*_LA4_ (*r* = −.17, *p* = .69), $\dot{Q}$ (*r* = .52, *p* = .19), or *SV* (*r* = .31, *p* = .46) at exhaustion (Figure 5). *S*_a_O_2_ and plasma lactate concentration during exercise in HYP were highly correlated with the change in run time of HYP after training (*S*_a_O_2_, *r* = .85, *p* = .0073; lactate, *r* = −.84, *p* = .0086), and *S*_a_O_2_ during exercise in HYP was also highly correlated with changes in $\dot{Q}$ (*r* = .72, *p* = .043) and *SV* (*r* = .77, *p* = .026) in HYP (Figure 6), whereas there were no correlations between these parameters in NOR (Figure 6).

## 4 DISCUSSION

The purpose of this study was to determine whether high‐intensity training in hypoxia would enhance aerobic capacity and exercise performance in Thoroughbred horses, and also to determine if individual horses would respond differently to normoxic and hypoxic training. The main findings of this study are that: (a) a 4 weeks training program, including 3 weekly high‐intensity hypoxic exercise bouts of only 2 min duration, improved run time, $\dot{V}$O_2_max, $\dot{Q}$ and, *SV* to a greater extent than the same training protocol under normoxic conditions in horses; (b) hypoxic training induced different training responses than normoxic training in exercise performance and aerobic capacity within a given horse; therefore, horses that did not respond well to the normoxic training protocol may adapt to training if exposed to an hypoxic training protocol; and (c) *S*_a_O_2_ during exercise in HYP was highly correlated with changes in run time, $\dot{Q}$ and *SV* at exhaustion in HYP after training, and we might be able to predict responders to hypoxic training from the response to acute high‐intensity training during exercise in hypoxia.

### 4.1 Key factors for the training adaptation to hypoxic training

Although LLTH has recently gained popularity in human athletes, an analysis of well‐controlled LLTH studies, including intermittent hypoxic training, failed to show greater improvements for sea‐level performance compared to the same training in normoxia (Faiss, Girard, & Millet, 2013). Vogt and Hoppeler (2010) stated that there appears to be no consistent benefit associated with the LLTH model among the reviewed studies, and there is no clear trend regarding LLTH as to differential effects of the severity of hypoxia or the duration of hypoxic exposure (Hoppeler, Klossner, & Vogt, 2008). Despite these negative outcomes of LLTH studies in humans, our study demonstrated that LLTH improved running performance (HYP, +28.4%; NOR, 10.4%), specific‐ $\dot{V}$O_2_max (HYP, +12.1%; NOR, +2.6%), and $\dot{Q}$ (HYP, 11.3%; NOR, −1.7%) to a greater extent than did normoxic training in horses.

These differences in the training effects of LLTH may be explained by the combination of the high training intensity (100% $\dot{V}$O_2_max) and moderate degree of hypoxia administered (15.0% inspired O_2_) in our study. Millet *et al*. (2010) stated that the combination of exercise duration and intensity, as well as the degree of hypoxia during training, are key factors in modulating the response to LLTH, and that greater responses occur with maximal or near‐maximal training interventions compared with submaximal training protocols. While Davie *et al*. (2017) failed to show any changes in heart rate and blood lactate concentration during a treadmill test after 6 weeks of moderate‐intensity training under hypoxic conditions (15% inspired O_2_) in horses, whereas, our another study has demonstrated that all‐out running for 2–3 min in hypoxia (15.1% inspired O_2_) twice a week for 3 weeks increased $\dot{V}$O_2_max (+8.9%) of well‐trained horses in which $\dot{V}$O_2_max had not increased over 3 consecutive weeks of supramaximal training in normoxia (Ohmura et al., 2017). These results suggest that exercise mode and intensity are also likely key factors for horses in mediating the response to the LLTH program, with higher training intensities appearing to be more beneficial than submaximal workloads as McLean *et al*. (McLean, Gore, & Kemp, 2014) indicated.

In this study, we observed higher cardiac output (+11.3% vs. PRE) and higher stroke volume (+5.3% vs. PRE) in HYP than those in NOR after training (Figure 3), but no changes in *C*_a‐v_O_2_ at exhaustion after training in both groups (Figure 4). $\dot{V}$O_2_ is expressed as the product of cardiac output ( $\dot{Q}$) and arterial‐mixed venous O_2_ difference (*C*_a‐v_O_2_; $\dot{V}$O_2_ = $\dot{Q}$ × *C*_a‐v_O_2_). These results indicate that the increase in O_2_ delivery, not skeletal muscle O_2_ extraction contributes to the changes in $\dot{V}$O_2_max after hypoxic training. These results are consistent with previous findings that $\dot{V}$O_2_max in horses changes proportionally to O_2_ supply as fraction of inspired O_2_ (F_I_O_2_) is varied to alter O_2_ supply, which suggests that $\dot{V}$O_2_max in horses is not limited by mitochondrial enzyme or substrate availability (Wagner et al., 1996).

### 4.2 Hemoglobin concentration after hypoxic training

Human athletes expect an increase of hemoglobin mass after LHTH and LHTL; however, most of the LLTH studies have failed to show additional increases in hemoglobin mass or concentration compared to normoxic training (Millet et al., 2010; Vogt & Hoppeler, 2010). Hemoglobin concentrations both at rest and at exhaustion did not change in both groups and were not different between the two groups in this study, probably because the exposure duration to hypoxia (approximately 3 min/day) was too short to enhance erythropoiesis, as previous studies in humans also demonstrated that hypoxic exposure only during exercise sessions is not sufficient to induce changes in hematologic parameters (Vogt & Hoppeler, 2010). Millet et al. (2010) reported that the minimum daily dose for stimulating erythropoiesis seems to be 12 hr/day.

### 4.3 Individual variability in response to hypoxic training

Exercise performance and/or training adaptation to hypoxia show large individual variations in human athletes (Chapman, 2013), and there is a potential need to identify responders and non‐responders to hypoxic training. However, Mounier *et al*. (2006) concluded that hypoxia inducible‐factor (HIF)‐1α gene expression in leukocytes after a 3‐hr hypoxia test performed before training did not predict poor and good responders to the LHTL model, and there is no previous literature on responders and non‐responders regarding the LLTH model as far as we know. Our results also demonstrate that the HYP protocol (i.e., the LLTH model) that differed only in the inspired O_2_ concentration from the NOR protocol, induced different training responses in run time, $\dot{V}$O_2_max, *V*_HRmax_, *V*_LA4_, $\dot{Q}$, and *SV* within a given horse (Figure 5). These findings suggest that horses that are not sensitive to the normoxic training protocol may experience training adaptation if exposed to the HYP training protocol. However, at the same time, these results also indicate that we cannot predict the training response within a given horse after HYP from the response after NOR, nor *vice versa*.

Chapman, Stager, Tanner, Stray‐Gundersen, & Levine (2011) reported that highly trained human athletes who are unable to maintain *S*_a_O_2_ during maximal exercise in normoxia are less able to maintain *S*_a_O_2_ in hypoxia and that these differences in response to hypoxia may lead to the differences seen in adaptation to hypoxic training. Therefore, we examined the relationship between blood gas variables during NOR and HYP exercise and the training adaptations after NOR and HYP. We found that horses that can maintain *S*_a_O_2_ during exercise in hypoxia showed greater improvements in run time, $\dot{Q}$ *,* and *SV* at exhaustion after 4 weeks of hypoxic training (Figure 6). We originally hypothesized that horses that experienced severe hypoxemia during HYP exercise would show greater improvements in exercise performance and aerobic capacity after hypoxic training, but the results of the present study found the opposite. We speculate that the hypoxic training (F_I_O_2_ 15%, 100% $\dot{V}$O_2_max 2 min, 3 sessions/week, 4 weeks) was too stressful for some horses that developed severe hypoxemia during hypoxic exercise and may have induced a state of overreaching. Vogt and Hoppeler (2010) stated that an important requisite for positive effects in hypoxic training is that the sessions do not overstress the athletes. The underlying mechanism responsible for these relationships is still unclear, and further study is needed to elucidate the mechanism responsible for this response, but we have the possibility to predict responders to HYP training from the response to acute high‐intensity exercise during HYP.

### 4.4 Lactate metabolism during hypoxia

Despite the fact that $\dot{V}$O_2_ during hypoxic exercise was lower than during NOR exercise and that the energy contribution to the glycolytic pathway increased to compensate for the decreased oxidative energy production during hypoxic exercise, plasma lactate concentrations during exercise in NOR and HYP were almost identical (19.3 vs. 19.5 mmol/l). Noakes (2009) has proposed that hypoxia may decrease central motor command during maximal exercise and thus reduces skeletal muscle recruitment, probably to protect the brain from severe hypoxemia. This phenomenon can partly explain the lower blood lactate concentrations than expected in HYP during exercise. *V*_LA4_ in the present study showed no significant difference between the NOR and HYP groups after 4 weeks of training, which seems reasonable given that there were no differences in plasma lactate concentration during exercise in NOR and HYP. However, Puype *et al*. (2013) reported that sprint interval training in moderate hypoxia (14.4% O_2_) for 6 weeks upregulated phosphofructokinase activity and power output at the *V*_LA4_ more than did sprint interval training in NOR. The effects of LLTH training on glycolytic metabolism are controversial, and further studies with different O_2_ concentrations, exercise intensities, and training durations are required to further elucidate the mechanisms involved.

## 5 CONCLUSION

We demonstrated that 4 weeks of high‐intensity training in normobaric hypoxia improved aerobic capacity and exercise performance in horses to a greater extent than did the same training program in normoxia. Furthermore, *S*_a_O_2_ during hypoxic exercise was highly correlated with changes in run time, $\dot{Q}$ *,* and *SV* at exhaustion after 4 weeks of hypoxic training. These factors have the potential to predict responders to hypoxic training. Our results can give a new insight into hypoxic training in horses and provide a new strategy for training programs in Thoroughbred racehorses.

## CONFLICT OF INTEREST

This study was funded by the Japan Racing Association. KM, HO, AM, HA, and TT are employees of the Japan Racing Association.

## AUTHOR CONTRIBUTIONS

Conceptualization: KM, HO, HA, and TT; Investigation: KM, HO, AM, HA, and TT; Formal analysis: KM and TT; Funding acquisition: HA; Methodology: KM, HO, and TT; Writing—original draft: KM; Writing—review & editing: HO, TT and JHJ.

6

**TABLE 1:** Parameters on aerobic capacity and blood gas analysis at normoxic incremental exercise tests before and after normoxic (NOR) and hypoxic (HYP) training

	NOR		HYP	
	Pre	Post	Pre	Post
*V*O_2_max (ml/(min kg))	162 ± 15	166 ± 13	160 ± 14	178 ± 8*
Speed_max_ (m/s)	12.6 ± 0.5	12.8 ± 0.5	12.4 ± 0.5	13.3 ± 0.7*
HR_max_ (bpm)	204 ± 9	210 ± 6	203 ± 9	214 ± 8
$\dot{Q}$ (ml/(s kg))	11.6 ± 0.8	11.4 ± 1.2	10.9 ± 0.8	12.1 ± 0.6*
*SV* (ml/kg)	3.43 ± 0.4	3.23 ± 0.4	3.24 ± 0.2	3.40 ± 0.2*
[*Hb*](g/dl)	23.4 ± 1.8	24.1 ± 1.5	23.5 ± 1.3	24.4 ± 1.7
*P*_a_O_2_ (mmHg)	81.5 ± 7.0	79.6 ± 4.4	80.7 ± 4.4	80.6 ± 3.5
*P*_v_O_2_ (mmHg)	22.4 ± 1.9	21.4 ± 1.3	22.5 ± 2.3	20.9 ± 3.2
A‐aDO_2_ (mmHg)	15.9 ± 5.8	15.7 ± 3.9	14.5 ± 4.1	14.6 ± 3.4
*P*_a_CO_2_ (mmHg)	53.1 ± 2.9	56.2 ± 4.6	52.5 ± 3.7	54.3 ± 6.0
*P*_v_CO_2_ (mmHg)	114 ± 17	118 ± 17	105 ± 15	125 ± 19
*C*_a_O_2_ (ml/dl)	27.2 ± 2.1	28.2 ± 1.6	27.5 ± 1.5	28.1 ± 2.0
*C*_v_O_2_ (ml/dl)	3.9 ± 0.9	4.0 ± 1.2	4.4 ± 1.1	3.5 ± 0.9
*S*_a_O_2_ (%)	85.8 ± 4.3	85.2 ± 4.8	87.4 ± 2.9	83.7 ± 2.9
*S*_v_O_2_ (%)	13.9 ± 3.2	12.9 ± 3.8	16.0 ± 3.9	10.9 ± 2.8*
pH_a_	7.192 ± 0.064	7.188 ± 0.097	7.223 ± 0.073	7.148 ± 0.049
pH_v_	7.077 ± 0.071	7.082 ± 0.082	7.126 ± 0.070	7.028 ± 0.052*
*LA*_peak_ (mmol/l)	23.1 ± 6.1	23.2 ± 7.0	20.7 ± 7.2	27.4 ± 5.7*

Note

Maximal oxygen consumption ( $\dot{V}$O_2_max), maximal treadmill speed attained (Speed_max_), maximal heart rate (HR_max_), cardiac output ( $\dot{Q}$), cardiac stroke volume (*SV*), Hemoglobin concentration ([*Hb*]), arterial and mixed‐venous O_2_ partial pressure (*P*_a_O_2_, *P*_v_O_2_), alveolar–arterial O_2_ difference (A‐aDO_2_), arterial and mixed‐venous carbon dioxide partial pressure (*P*_a_CO_2_, *P*_v_CO_2_), arterial and mixed‐venous O_2_ concentration (*C*_a_O_2_, *C*_v_O_2_), arterial and mixed‐venous O_2_ saturation (*S*_a_O_2_, *S*_v_O_2_) and arterial and mixed‐venous pH (pH_a_, pH_v_) at exhaustion, and peak plasma lactate concentration (*LA*_peak_). Values are means ± *SD* for eight horses.

* Significantly different from NOR (*p* < .05).
